# The complete genome sequence of the African buffalo (*Syncerus caffer*)

**DOI:** 10.1186/s12864-016-3364-0

**Published:** 2016-12-07

**Authors:** Brigitte Glanzmann, Marlo Möller, Nikki le Roex, Gerard Tromp, Eileen G. Hoal, Paul D. van Helden

**Affiliations:** SA MRC Centre for TB Research, DST/NRF Centre of Excellence for Biomedical TB Research, Division of Molecular Biology and Human Genetics, Faculty of Medicine and Health Sciences, Stellenbosch University, Cape Town, South Africa

**Keywords:** Genome assembly, *Syncerus caffer*, *Bos taurus*, Genetic diversity

## Abstract

**Background:**

The African buffalo *(Syncerus caffer)* is an important role player in the savannah ecosystem. It has become a species of relevance because of its role as a wildlife maintenance host for an array of infectious and zoonotic diseases some of which include corridor disease, foot-and-mouth disease and bovine tuberculosis. To date, no complete genome sequence for *S. caffer* had been available for study and the genomes of other species such as the domestic cow *(Bos taurus)* had been used as a proxy for any genetics analysis conducted on this species*.* Here, the high coverage genome sequence of the African buffalo *(S. caffer)* is presented.

**Results:**

A total of 19,765 genes were predicted and 19,296 genes could be successfully annotated to *S. caffer* while 469 genes remained unannotated. Moreover, in order to extend a detailed annotation of *S. caffer*, gene clusters were constructed using twelve additional mammalian genomes. The *S. caffer* genome contains 10,988 gene clusters, of which 62 are shared exclusively between *B. taurus* and *S. caffer*.

**Conclusions:**

This study provides a unique genomic perspective for the *S. caffer*, allowing for the identification of novel variants that may play a role in the natural history and physiological adaptations.

**Electronic supplementary material:**

The online version of this article (doi:10.1186/s12864-016-3364-0) contains supplementary material, which is available to authorized users.

## Background

The African buffalo (*Syncerus caffer*) is the largest bovid species in the African savannah ecosystem. Buffalo are of great ecological importance because of their role as bulk feeders in the grazing hierarchy. Due to their size they are able to process taller and coarser grasses than most other species [1], playing an important facilitative role for the smaller grazers [2]. They inhabit zones with almost all vegetation types, provided a permanent water source is present. In addition, they are an important prey species and have high economic value in the ecotourism and hunting industries [3]. The African buffalo also hosts a vast array of nematodes, pathogens and infectious diseases and plays an important role in the maintenance and transmission of economically important livestock diseases such as foot-and-mouth disease (FMD), bovine brucellosis, corridor disease and bovine tuberculosis (BTB) [4, 5]. For numerous other diseases, buffalo may act as amplifier or incidental hosts, as is the case with ehrlichiosis, Rift Valley fever and anthrax [6]. It is assumed that the African buffalo, unlike some domestic bovids (e.g. cattle), may exhibit partial resistance to some of these diseases, highlighting the importance of understanding the genetic mechanisms at work.

Advances in methods for characterizing the genetic variation in individuals, populations and species have revolutionized ecological research. Population genetic diversity, inbreeding, hybridization, species designations, dispersal patterns and evolutionary processes are just a few of the applications of genetic data in the conservation and management of wildlife [7, 8]. One of the greatest challenges of working with non-model species is the lack of availability of genome variation data with which to design these studies [9]. The assembly of an accurate genome for important non-model study species provides an invaluable resource for research. Complete and accurate reference genome information prevents the erroneous identification of polymorphisms, and misalignments [10]. In the absence of a complete reference genome, a related species can be used as a proxy reference to facilitate the identification of various single nucleotide variants (SNVs), but the amount of sequence that can be accurately mapped may be insignificant [11].

The identification of novel genetic variants in an important species such as the African buffalo may help to provide answers to numerous genetic and other research questions, including disease susceptibility, that have heretofore been unattainable [12]. Previously, we aligned buffalo short reads obtained from high-throughput sequencing to the *B. taurus* genome assembly to facilitate SNV discovery [13]. Here, we present the first complete *de novo* assembled full-length genome for the African buffalo with an assembled N50 contig size reaching 43 kilobase pairs (kbp) and an N50 scaffold length of 2.4 Mb, which represents the first fully sequenced and *de novo* assembled sequence of the African buffalo. We carried out a number of additional analyses, including evolutionary analyses and genetic content. Our results will significantly aid in understanding the genetics of the African buffalo and contribute to the fields of molecular ecology, population genetics and disease susceptibility, ultimately supporting conservation and management efforts.

## Results

Twelve mate-pair libraries with different insert sizes were prepared using DNA from a 2-year old male buffalo and sequenced to a high (60-fold) coverage on an Illumina Hi-Seq 2000 and assembled *de novo*. Following data filtration and the removal of low quality reads, a total of 242.39 Gbp of usable sequence (equating to 89.78-fold coverage of the whole genome) and an average read length of 78.67 bp was obtained (Additional file 1: Table S1). The total length of the genome assembly equated to 2.68 Gbp and a total of 1235 scaffold contigs made up 90% of the genome assembly and 97.9% of the estimated length (Additional file 2: Table S2). It was estimated that the genome size of *S. caffer* is 2732 Mbp (Additional file 3: Figure S1). A total of 19,765 genes were predicted and of these a total of 19,296 genes were annotated to *S. caffer* while a total of 469 genes could not be annotated (Table 1). Data analysis and annotation of the non-coding RNA identified a large number of miRNA and tRNA (31,940 and 36,163 respectively). The estimated heterozygosity ratio of the sequenced buffalo was 0.6 × 10^−3^ (Additional file 4: Figure S2) and we estimated 1,639,766 heterozygous SNVs.

**Table 1:** Assembly and annotation of the *S. caffer* genome

Feature		Size	Source
SOAP *de novo* assembly		--	Supplementary Table 1
Estimated genome size (assembly and 17mer)		2732 Mb	--
N50 contigs		43. kbp	Supplementary Table 2
N50 scaffolds		2.4 Mb	Supplementary Table 2
Average GC content		0.417	Supplementary Table 2
Coding genes	a. 19,296 annotated	430.18 Mb	--
	b. 469 unannotated		
Non-coding RNA (70,595 loci)	a. 31,940 micro RNA	3.25 Mb	Data not shown
	b. 1593 small nuclear RNA	184.60 kbp	
	c. 36,163 transport RNA	2.64 Mb	
	d. 899 ribosomal RNA	93.94 kbp	
Repetitive elements (37.21%)	Tandem repeats	972.19 Mb	Supplementary Table 3, 4, 5

**Fig. 1:**
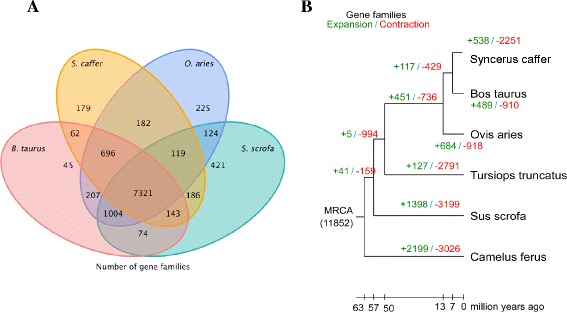
Analysis of orthologous gene families. **a**. Unique and shared gene families between the *S. caffer* genome and other species. **b**. Evolution of ortholog gene clusters. The estimated numbers of ortholog groups in the common ancestral species are shown in the internal nodes. The numbers of orthologous groups that have expanded or contracted in each lineage are shown on the corresponding branch, with + referring to expansion and – referring to contraction. The *S. caffer* genome has 10,988 orthologous gene families and a total of 7321 are shared among four species. A total of 62 are shared exclusively between *S. caffer* and *B. taurus* and 179 predicted genes that are unique to *S. caffer.* Both figures were based on the comparison of orthologous gene families among thirteen mammalian species

To extend a detailed annotation, the *S. caffer* genome, gene clusters were constructed using twelve additional mammalian genomes (*Felis catus, Rattus norvegicus, Pan troglodytes, Canis familiaris, Equus caballus, H. sapiens, S. scrofa, O. aries, T. truncatus, B. taurus, Copelatus ferus* and *M. musculus*). The *S. caffer* genome contains 10,988 orthologous gene clusters and a total of 7321 are shared among four species (Table 2; Fig. 1a). A total of 62 predicted gene clusters are shared exclusively between *S. caffer* and *B. taurus* and 179 are unique to *S. caffer* (Fig. 1a). It was determined that the divergence time between *S. caffer* and *B. taurus* is 5.7–9.3 million years ago (MYA) (Additional file 5: Figure S5).

**Table 2:** Summary of gene families of *S. caffer* and twelve other mammalian genomes

Species	Total number of orthologous genes	Number of unclustered genes	Number of gene families	Number of unique families	Average number of genes per family
*F. catus*	19,440	613	9549	14	1.97
*R. norvegicus*	22,656	432	9698	22	2.29
*P. troglodytes*	18,613	484	9580	25	1.89
*C. familiaris*	19,818	771	9508	16	2.00
*E. caballus*	20,372	229	9364	22	2.15
*H. sapiens*	22,214	371	9836	24	2.22
*M. musculus*	22,484	655	9718	45	2.25
*S. scrofa*	21,526	1860	9392	120	2.09
*O. aries*	20,786	523	9878	29	2.05
*T. truncatus*	16,476	245	9082	2	1.79
*B. taurus*	19,950	108	9552	1	2.08
*S. caffer*	**19,292**	**1295**	**8888**	**52**	**2.02**
*C. ferus* ^*a*^	23,017	784	9269	16	2.40

^a^ *C. ferus* genome was downloaded from NCBI(ftp.ncbi.nih.gov/genomes/Camelus_ferus), while all other genomes were download from Ensemble release-78(ftp.ensembl.org/pub/release-78)

The figures in bold are intended to highlight the information obtained from the buffalo

To investigate signatures of selection we obtained a total of 2236 1:1 orthologous gene sets in *S. caffer*, *B. taurus, C. ferus, O. aries, S. scrofa* and *T. truncates* and this was based on the gene family results. Finally, we inferred 120 genes which contain positive selected sites in buffalo. These include ubiquitin carboxyl-terminal hydrolase 26 (*UCHL26*)*,* Interleukin 19 (*IL19*) and Cyclin B (*CCNB*). Based on the comparison of orthologous gene families among the 12 mammalian species, the *S. caffer* genome has 538 expanded and 2251 contracted gene families when compared to *B. taurus* as a common ancestor (Fig. 1b). The expanded genes were coupled to a large variety of GO terms, including G-coupled protein and olfactory receptors.

## Discussion

The African buffalo has become a species of great interest in recent years because it serves as maintenance host for numerous infectious and zoonotic diseases such as FMD, corridor disease and bovine tuberculosis [5]. In addition, its high economic value in the ecotourism and trophy hunting industries make this species invaluable to game ranchers and breeders [4, 5, 14]. The African buffalo is one of only three main species of buffalo found in the world. The other two include the American Bison (*Bison bison)* and the domesticated Asiatic buffalo (*Bubalus bubalis*). Reference genomes of 2.82 Gbp and 2.77 Gbp have been assemble d for these respectively, but are not publically available [15, 16]. The *Bubalus bubalis* genome was found to encode 21,550 protein coding genes, which is comparable to the African buffalo genome we report here, which is 2.73 Gbp in size with a total of 19,765 predicted protein coding genes and of these 19,296 were annotated. Moreover, the annotation of non-coding RNA identified unexpectedly large numbers of miRNA and tRNA [17, 18]. The use of standard tools namely tRNAscanner SE (version 1.23) as well as alignment using BLAST and INFERNAL for *de novo* non-coding RNA annotation consistently identified 31,940 miRNA genes. The large number is implausible (data not shown), suggesting the need for improved prediction tools in some species including *S. caffer*.

Although big game species such as the African lion, African elephant and the white rhino have been sequenced [19–21], the only suitable reference genome publically available for the alignment of African buffalo sequences was that of *B. taurus* [13]. *S. caffer* has 52 chromosomes [22] compared to the 30 chromosomes found in *B. taurus* [23]. Our analyses here estimated 1,639,766 heterozygous SNVs in the sequenced buffalo, compared to the 3,833,249 heterozygous variants identified in the Holstein *B. taurus* genome [24]. The African buffalo is not an antecedent of the cow and their most recent common ancestor is estimated to have existed approximately 5 - 10 MYA, at the time of divergence of the sub-tribes Bubalina, which is composed of the *Syncerus* and *Bubalus* genera and Bovina, comprising the *Bos* and *Bison* genera [25, 26]. In previous work, we determined that only 19 to 23% of the low coverage buffalo short reads mapped to the cow reference genome using BWA and Bowtie, illustrating the need for a species-specific reference [13].

We anticipate that the annotated African buffalo genome will facilitate our future genetic association studies of susceptibility to BTB, which is a threat to conservation areas in South Africa [27]. Previously, we identified novel SNVs by Sanger sequencing conserved regions across species, a process that was time-consuming and often resulted in non-specific amplification [28]. Alternatively, we had to rely on short reads mapped to an unsuitable reference genome [13]. It will now be possible to design species-specific primers for susceptibility genes of interest, based on the gene annotation completed here. Low coverage genome sequencing of additional buffalo will allow us to establish a SNV database, which would also be a resource for future population genetic and disease association studies [29].

Several of the genes that were substantially expanded and contracted in the African buffalo compared to other mammals are involved in immunity. This includes the contracted genes Chemokine (C-X-C motif) ligand 2 (*CXCL2*) and complement component 8 alpha subunit (*C8A*) as well as the expanded genes T cell receptor gamma variable 3 (*TRGV3*) and Killer immunoglobulin-like receptor KIR3DL splice variant 3 (*KIR3DL*). In total it is estimated that there are 175 genes involved in immune responses in the African buffalo and these are possible candidate genes to investigate in disease susceptibility studies.

## Conclusions

In summary, the African buffalo genome offers unique insight into the phylogenetic history and adaptation of an ecologically important species. Additionally, the availability of a complete reference genome allows for improved mapping of short reads, thereby aiding in novel SNV discovery and future genetic studies.

## Methods

Buffalo blood was obtained from a number of *S. caffer* individuals from the southern section of the Kruger National Park, South Africa for other projects. Based on the quantity and quality of the DNA available and to meet the sequencing requirements, the DNA from a 2-year old male was chosen for DNA sequencing. DNA was extracted from whole blood using the salt-chloroform extraction method [30]. No ethics approval was required as the DNA was collected and extracted for a previous study, under the directive of South African National Parks (SANParks), and its use in the present study is incidental.

### Genome assembly for *S. caffer*

All libraries were sequenced using an Illumina Hi-Seq 2000 instrument. The sequencing libraries were constructed with insert sizes of 170 bp, 500 bp, 800 bp, 2 kbp, 5 kbp and 10 kbp respectively. *S. caffer* genome scaffolds (1235 scaffolds; N50 contig: 43 kbp, N50 scaffold: 2.4 Mb) were aligned to the reference *B. taurus* genome assembly. The protein coding genes in *S. caffer* were determined through the integration of annotations from homology-based methods as well as *de novo* gene assembly. For homology-based prediction, proteins from *B. taurus, Homo sapiens, Mus musculus, Ovis aries, Sus scrofa, Tursiops truncatus* were mapped to the *S. caffer* genome using TblastN [31] and were then submitted to GeneWise version 2.2.0 [32] in order to obtain gene models. For *de novo* prediction, two software programs were used: SNAP [33] and AUGUSTUS [34].

### Genome size evaluation

The genome size of an individual can be estimated from the K-mer frequency of the read data. Importantly, the K should be large enough that most of the genome can be distinguished. For most eukaryotic genomes, a K-mer value of 17 is used (K = 17). For the present study, a total of 74 Gb (approximately 30X) of the data could be retained for 17-mer analyses. Simulations were done to estimate the heterozygosity ratio.

### Genome assembly

The short reads were assembled using the latest version of SOAPdenovo (http://soap.genomics.org.cn/soapdenovo.html), a genome assembler that has been developed for specific use with next generation short read sequences [35]. SOAPdenovo uses the de Bruijn graph algorithm, which is sensitive to sequencing errors and for this reason, only high-quality, filtered data were used for the *de novo* assembly. Gaps in the initial assembly were filled using Gapcloser [35]. Short reads from fragmented small insert-size libraries (<500 bp) were assembled into contigs using sequence overlap information. Contigs were not extended into regions in which repeat sequences created ambiguous associations. The resulting assembly contained a small contig N50 with a length of 43 kbp and a scaffold N50 length of 2.4 Mbp.

### GC content and sequencing depth analysis

The completeness of the genome assembly was evaluated by aligning sequence reads to the newly generated genome assembly and subsequently determining the percentage of total aligned reads. High quality reads i.e. reads with a percentage of high quality bases that satisfies a user-specific cutoff, with an average coverage of 38-fold were aligned to the *S. caffer* genome using BWA-MEM [36]. A total of 99.93% reads were aligned to the assembly.

Variable GC content difference is a primary determinant for the non-random distribution of sequencing depth [37]. Moreover, the distribution of GC content vs. sequencing depth is a means of ascertaining sequencing bias or contamination (Additional file 6: Figure S3 and Additional file 7: Figure S4). Regions with very low GC contents (<20%) or very high GC contents (>80%) will have a low sequencing depth. Should specific regions of the genome have a GC content that is significantly different from that predicted, it is reasonable to assume that there may be bacterial, viral or fungal contamination in the sample and reads should be eliminated by additional alignment. For the purposes of this study, two sequencing depth distribution blocks were obtained; one with an average sequencing depth of 38X and the other with a sequencing depth of 19X. The sample that was sequenced was a male, and it is anticipated that hemizygous regions will have lower coverage depth because there is only one allelic counterpart on the Y chromosome. A total of 2825 sequences are found at low coverage (19X) (Additional file 7: Figure S4) and were subsequently aligned to the X and Y chromosomes of *B. taurus*. It was found that 99.85% of the sequences align directly to the X and Y chromosome of *B. taurus* while 0.6 and 0.64% align to the fungi and bacterial databases respectively. It was therefore concluded that this block of low coverage region forms part of the sex chromosomes and would thus not adversely affect the *de novo* assembly.

### Gene annotation

1. Repetitive element annotation
  Tandem Repeats Finder (TRF) was used to identify non-interspersed repetitive elements. Transposable elements (TEs) were predicted in the homology searches found in Repbase TE libraries using Repeat ProteinMask and RepeatMasker (Additional file 8: Table S3, Additional file 9: Table S4 and Additional file 10: Table S5).
2. Non-coding RNA annotation
  A total of four non-coding RNA (ncRNA) types were annotated.
  micro RNA (miRNA)
  transfer RNA (tRNA)
  ribosomal RNA (rRNA)
  small nuclear RNA (sn-RNA)
  All four of these RNA types were found in *S. caffer* genome using the complete genome sequence. Scanning for tRNA was performed using tRNAscan-SE [38] by using a short interspersed elements (SINEs) premasked genome to search for reliable tRNA positions. The snRNAs and miRNAs were identified by aligning with BLAST and INFERNAL to search for putative sequences in the Rfam database [39]. The rRNA fragments were identified by aligning the rRNA template sequences from the human genome using BlastN.
3. Gene prediction
  The protein coding genes in *S. caffer* were determined through the integration of annotations from homology-based methods as well as *de novo* gene assembly. For homology-based prediction, proteins from *B. taurus, H. sapiens, M. musculus, O. aries, S. scrofa, T. truncates* and mapped to the buffalo genomes using TblastN [31] and were then submitted to GeneWise [32] in order to obtain gene models. For *de novo* prediction, two software programs were used: SNAP [33] and Augustus [34] with gene model parameters trained from *H. sapiens*, and filtered partial genes and small genes that had less than 150 bp coding length. This followed with the alignment of the predictions to a TE protein database using BlastP [31] with an E-value ≤ 1e-5 and filtered TE-derived genes that had more than 50% alignment rate (Additional file 11: Table S6)*.*

### Gene family construction

To determine the genetic evolution in *S. caffer*, gene cluster analysis included the genomes of 13 mammals. Proteins of all genes for each of the species chosen were analyzed using Treefam [40]. All proteins sequences were aligned to themselves using BlastP [31] with E-value cut-off of 1e-7 (Table 2).

### Phylogeny and divergence

A total of 1745 single copy gene clusters were identified and were used to construct a phylogenetic tree of the 13 mammal species. MUSCLE [3] was used for alignment and gaps removed by Gblocks [41]. A total of 2,265,138 (55.45%) remained and were used to construct the phylogeny. Moreover, the species divergence time was estimated based on 195,689 fourfold degenerate sites via Bayesian estimation approach using PAML [42]. The phylogenetic relationship of *S. caffer* and twelve additional mammals was predicted. The data can be accessed using the following link: http://purl.org/phylo/treebase/phylows/study/TB2:S20207?x-access-code=f3bdb11f9f55ac0609abf60ab1c01255&format=html.

### Gene family expansion and contraction

The evolutionary changes in the protein family size (expansion or contraction) were analyzed using the CAFÉ program [43]. This package assesses the protein family expansion or contraction based on the topology of the phylogenetic tree (Fig. 1).

### Branch site positive selection

Orthologues were aligned using the PRANK alignment algorithm available the GUIDANCE software program [44], which can improve the performance of positive selection inference by filtering out unreliable alignment regions.

### Ka/Ks of *S. caffer* and *B. taurus*

The ratio of the total number of non-synonymous substitutions per non-synonymous site (Ka) to the number of synonymous substitutions per synonymous site (Ks), referred to as Ka/Ks, can be used as an indicator of selective pressure acting on a protein-coding gene. In total, 19,994 orthologues from both *S. caffer* and *B. taurus* were chosen using Reciprocal Best Hits (RBH) methodology [45] based on the BLAST alignment. Subsequently, the coding regions of the two species were aligned using webPRANK [46] and unreliable regions were removed. Ka/Ks scores were calculated using the KaKs Calculator [47].

## Additional files

Additional file 1: Table S1. Data statistics for de novo assembly of the *S. caffer* genome. (PDF 63 kb)
Additional file 2: Table S2. Genome assembly results. (PDF 62 kb)
Additional file 3: Figure S1. 17-mer coverage distribution. (PDF 69 kb)
Additional file 4: Figure S2. Simulations to estimate heterozygosity ratio. (PDF 72 kb)
Additional file 5: Figure S5. Phylogeny and divergence of 13 mammals. (PDF 352 kb)
Additional file 6: Figure S3. Distribution of the read depth for the *de novo* assembled *S. caffer* genome. (PDF 86 kb)
Additional file 7: Figure S4. GC content and average sequencing depth values for the *S. caffer* genome. (PDF 361 kb)
Additional file 8: Table S3. Summary of repetitive elements obtained using 3 pipelines. (PDF 46 kb)
Additional file 9: Table S4. Summary of TE classification. (PDF 49 kb)
Additional file 10: Table S5. Top 10 copy number variations TE subfamily in African buffalo genome. (PDF 50 kb)
Additional file 11: Table S6. Summary of function annotation for African buffalo. (PDF 45 kb)

## Abbreviations

BTB: Bovine tuberculosis
FMD: Foot and mouth disease
kbp: kilobase pairs
MYA: Million years ago
RBH: Reciprocal best hits
SANParks: South African National Parks
SINE: Short interspersed elements
SNVs: Single nucleotide variants
TE: Transposable elements
TRF: Tandem repeats finder.
