# Supplementary material for: The complete genome sequence of the African buffalo (Syncerus caffer)
Source: BMC Genomics. 2016 Dec 7;17:1001. doi: 10.1186/s12864-016-3364-0 (PMC5142436; doi:10.1186/s12864-016-3364-0)
Supplement: Additional file 1: Table S1. — Data statistics for de novo assembly of the S. caffer genome. (PDF 63 kb) [file 12864_2016_3364_MOESM1_ESM.pdf]

Supplementary Table 1: Data statistics for *de novo* assembly of the *S. caffer* genome

Insert Size (bp)	Number of Libraries	Read Length (bp)	Data available after filtration (Gigabases)	Coverage
170	1	100_100	38.81	14.37
500	2	100_100	65.41	24.23
800	2	125_125	77.29	28.63
2,000	3	49_49	28.17	10.43
5,000	2	49_49	14.65	5.42
10,000	2	49_49	18.08	6.70
**TOTAL**	**12**	**--**	**242.39**	**89.78**
