# Supplementary material for: The complete genome sequence of the African buffalo (Syncerus caffer)
Source: BMC Genomics. 2016 Dec 7;17:1001. doi: 10.1186/s12864-016-3364-0 (PMC5142436; doi:10.1186/s12864-016-3364-0)
Supplement: Additional file 2: Table S2. — Genome assembly results. (PDF 62 kb) [file 12864_2016_3364_MOESM2_ESM.pdf]

Supplementary Table 2: Genome assembly results

	Scaffold		Contig	
	**Length (bp)**	**Number**	**Length (bp)**	**Number**
**Maximum length**	16,927,952	--	471,476	--
**N10**	7,037,875	30	118,985	1,669
**N20**	5,014,521	76	86,229	4,285
**N30**	3,945,199	136	67,095	7,736
**N40**	3,102,229	213	53,686	12,106
**N50**	**2,411,048**	**312**	**42,601**	**17,587**
**N60**	1,898,631	438	33,401	24,516
**N70**	1,409,435	604	25,193	33,503
**N80**	946,337	833	17,424	45,902
**N90**	421,185	1,235	9,166	66,033
**Total length**	2,688,614,675	--	2,612,853,568	--
**Number ≥ 100 bp**	--	442,401	--	561,609
**Number ≥ 2000 bp**	--	5,350	--	96,566
**Average GC content**	0.406	--	0.417	--
