# Supplementary material for: The complete genome sequence of the African buffalo (Syncerus caffer)
Source: BMC Genomics. 2016 Dec 7;17:1001. doi: 10.1186/s12864-016-3364-0 (PMC5142436; doi:10.1186/s12864-016-3364-0)
Supplement: Additional file 3: Figure S1. — 17-mer coverage distribution. (PDF 69 kb) [file 12864_2016_3364_MOESM3_ESM.pdf]

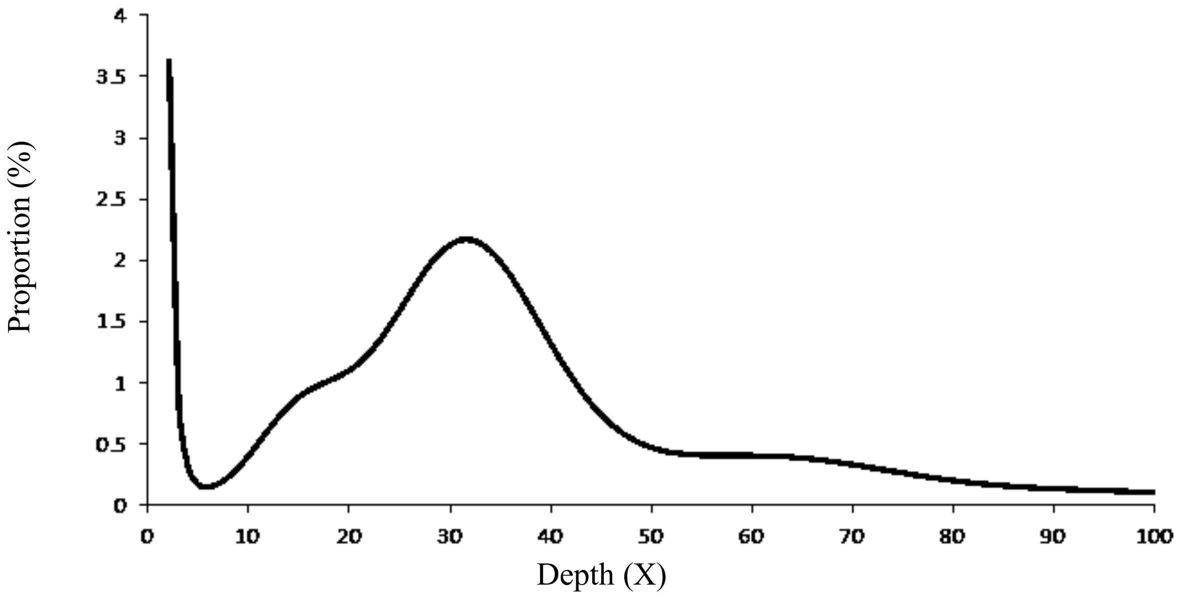

Supplementary Figure 1: 17-mer coverage distribution. The distribution was used to estimate the *S. caffer* genome size. The x-axis is 17-mercoverage depth and the y-axis is the percentage of the total number of 17-mers.
