# Supplementary material for: The complete genome sequence of the African buffalo (Syncerus caffer)
Source: BMC Genomics. 2016 Dec 7;17:1001. doi: 10.1186/s12864-016-3364-0 (PMC5142436; doi:10.1186/s12864-016-3364-0)
Supplement: Additional file 4: Figure S2. — Simulations to estimate heterozygosity ratio. (PDF 72 kb) [file 12864_2016_3364_MOESM4_ESM.pdf]

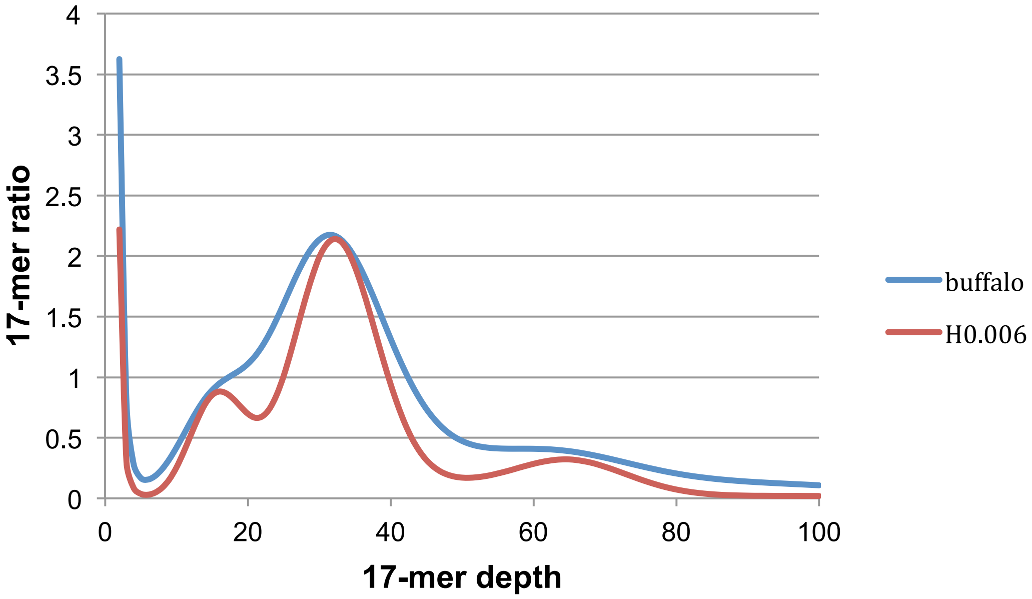

Supplementary Figure 2: Simulations to estimate heterozygosity ratio. The X axis is the depth of 17-mer and the Y axis is the ratio of 17-mer. The H_0.006 indicates that the heterozygosity ratio is 0.6%.
