# Supplementary material for: The complete genome sequence of the African buffalo (Syncerus caffer)
Source: BMC Genomics. 2016 Dec 7;17:1001. doi: 10.1186/s12864-016-3364-0 (PMC5142436; doi:10.1186/s12864-016-3364-0)
Supplement: Additional file 5: Figure S5. — Phylogeny and divergence of 13 mammals. (PDF 352 kb) [file 12864_2016_3364_MOESM5_ESM.pdf]

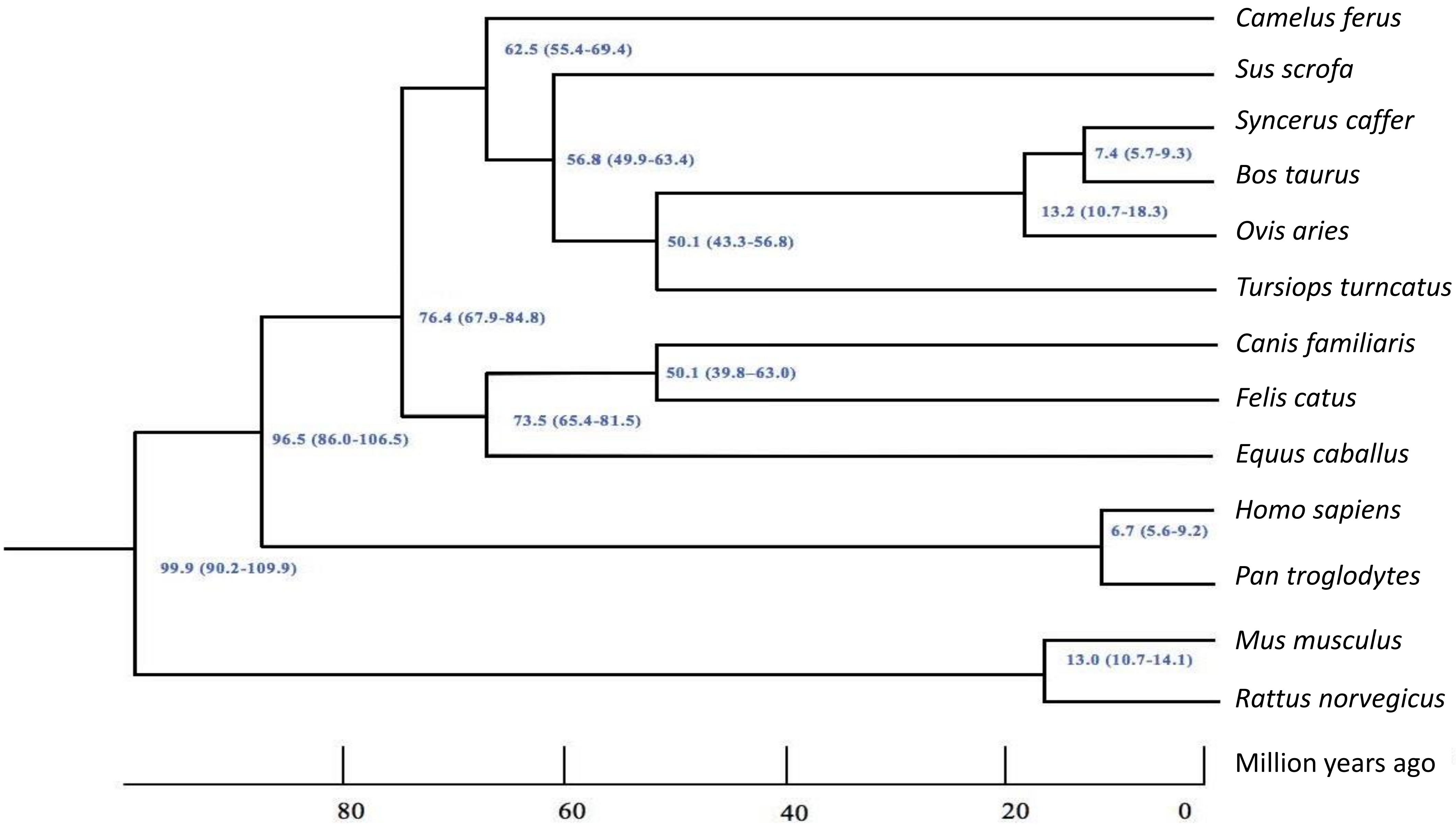

Supplementary Figure 5: Phylogeny and divergence time of thirteen mammals. It was determined that *S. caffer* and *B. taurus* diversified approximately 7.4 million years ago. The figures in brackets indicate the highest posterior density (HPD) credibility values.
