# Supplementary material for: The complete genome sequence of the African buffalo (Syncerus caffer)
Source: BMC Genomics. 2016 Dec 7;17:1001. doi: 10.1186/s12864-016-3364-0 (PMC5142436; doi:10.1186/s12864-016-3364-0)
Supplement: Additional file 6: Figure S3. — Distribution of the read depth for the de novo assembled S. caffer genome. (PDF 86 kb) [file 12864_2016_3364_MOESM6_ESM.pdf]

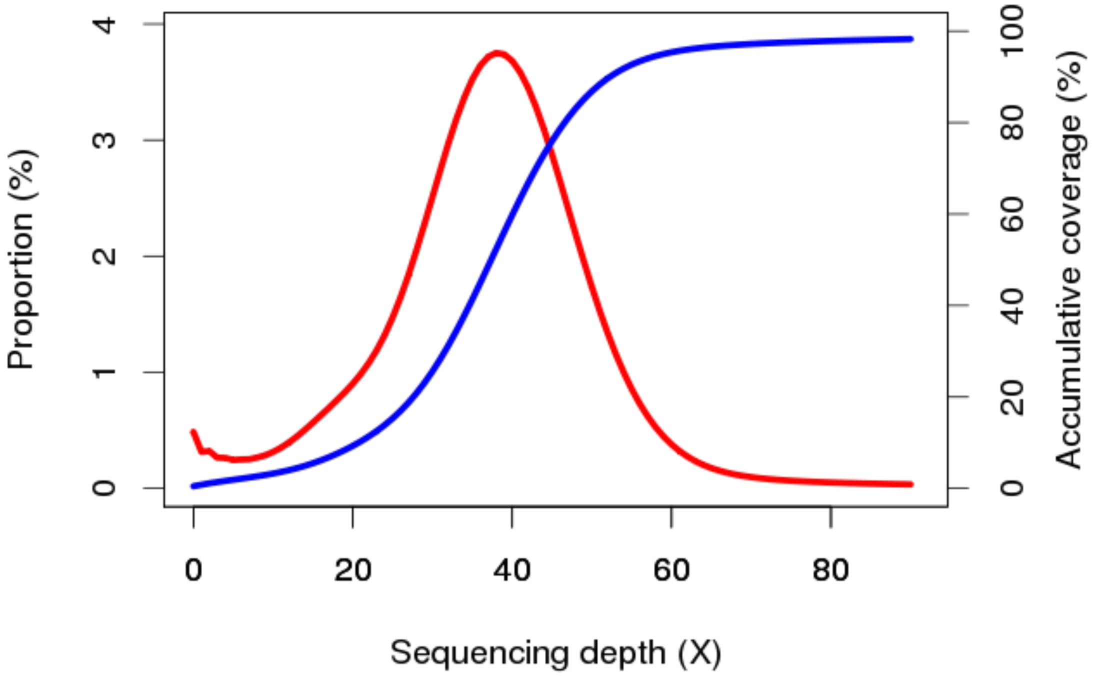

Supplementary Figure 3: Distribution of the read depth for the *de novo* assembled *S. caffer* genome. The x-axis is the number of reads aligned to a nucleotide position; the y-axis is the percentage of such genome positions. The mean sequencing depth is 38X.
