# Supplementary material for: The complete genome sequence of the African buffalo (Syncerus caffer)
Source: BMC Genomics. 2016 Dec 7;17:1001. doi: 10.1186/s12864-016-3364-0 (PMC5142436; doi:10.1186/s12864-016-3364-0)
Supplement: Additional file 7: Figure S4. — GC content and average sequencing depth values for the S. caffer genome. (PDF 361 kb) [file 12864_2016_3364_MOESM7_ESM.pdf]

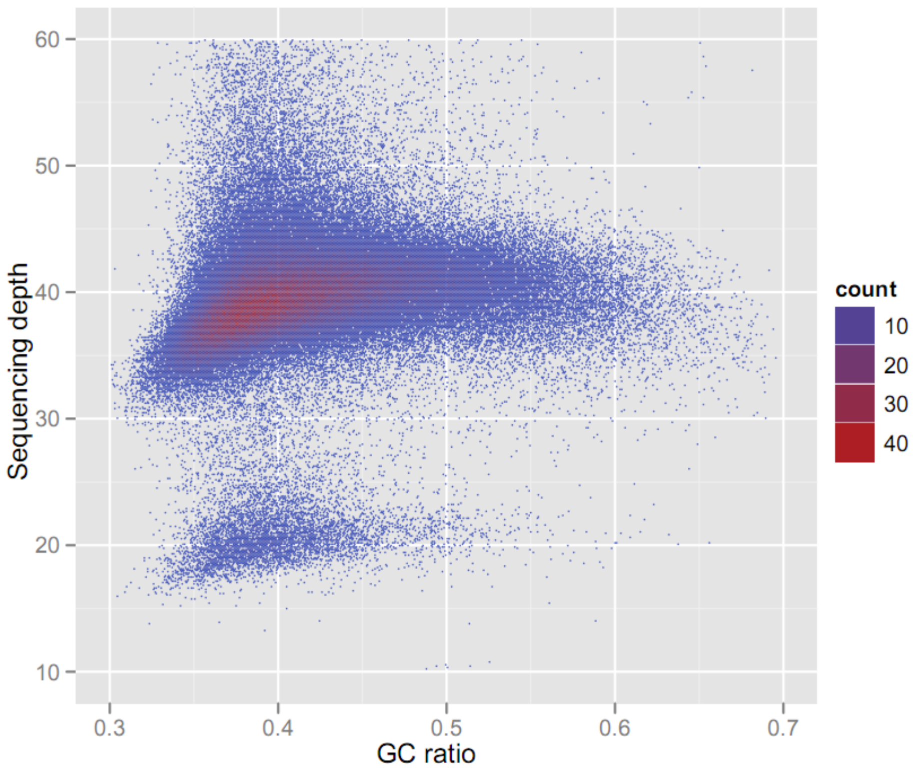

Supplementary Figure 4: GC content and average sequencing depth values for the *S. caffer* genome. The x and y-axes represent the GC content and average depth values respectively. The dominant GC content value lies within 35-40%. The subgroup (the lower cloud with depth 18-25 and lower GC content) is a representation of the sex chromosomes.
