# Supplementary material for: The complete genome sequence of the African buffalo (Syncerus caffer)
Source: BMC Genomics. 2016 Dec 7;17:1001. doi: 10.1186/s12864-016-3364-0 (PMC5142436; doi:10.1186/s12864-016-3364-0)
Supplement: Additional file 8: Table S3. — Summary of repetitive elements obtained using 3 pipelines. (PDF 46 kb) [file 12864_2016_3364_MOESM8_ESM.pdf]

Supplementary Table 3: Summary of repetitive elements obtained using 3 pipelines

Software	Number of bases	Percentage of genome
Repeatmasker	931,634,084	35.66
RepeatProteinMask	457,776,102	17.52
TRF	36,797,712	1.41
Combined	972,191,200	37.21
