# Supplementary material for: The complete genome sequence of the African buffalo (Syncerus caffer)
Source: BMC Genomics. 2016 Dec 7;17:1001. doi: 10.1186/s12864-016-3364-0 (PMC5142436; doi:10.1186/s12864-016-3364-0)
Supplement: Additional file 9: Table S4. — Summary of TE classification. (PDF 49 kb) [file 12864_2016_3364_MOESM9_ESM.pdf]

Supplementary Table 4: Summary of TE classification

	RepeatMasker		ProteinMasker		Combined	
TE_subtype	Number of bases	Percentage of genome	Number of bases	Percentage of genome	Number of bases	Percentage of genome
DNA	32,230,438	1.23	6,688,000	0.26	32,888,317	1.26
LINE	642,750,230	24.60	440,271,146	16.85	668,187,916	25.57
LTR	91,056,756	3.48	11,035,446	0.42	94,160,822	3.60
SINE	182,590,819	6.99	0	0.00	182,590,819	6.99
Other	417	0.00	0	0.00	417	0.00
