# Supplementary material for: The complete genome sequence of the African buffalo (Syncerus caffer)
Source: BMC Genomics. 2016 Dec 7;17:1001. doi: 10.1186/s12864-016-3364-0 (PMC5142436; doi:10.1186/s12864-016-3364-0)
Supplement: Additional file 10: Table S5. — Top 10 copy number variations TE subfamily in African buffalo genome. (PDF 50 kb) [file 12864_2016_3364_MOESM10_ESM.pdf]

Supplementary Table 5: Top 10 copy number variations TE subfamily in African buffalo genome

**TE classification**	**Copy number**	**Number of bases**	**Percentage**
LINE/RTE	1,264,674	297,392,318	11.38
LINE/L1	657,584	149,766,018	5.73
LTR/ERV1	28,120	6,039,131	0.23
DNA/TcMar	18,117	4,443,966	0.17
LTR/ERVL	13,234	3,225,152	0.12
DNA/hAT	7,858	2,140,904	0.08
LINE/L2	7,408	2,771,872	0.11
LTR/ERVK	7,348	1,834,103	0.07
LINE/CR1	6,284	2,616,580	0.10
LINE/R2	5,059	2,127,895	0.08
