# Supplementary material for: The complete genome sequence of the African buffalo (Syncerus caffer)
Source: BMC Genomics. 2016 Dec 7;17:1001. doi: 10.1186/s12864-016-3364-0 (PMC5142436; doi:10.1186/s12864-016-3364-0)
Supplement: Additional file 11: Table S6. — Summary of function annotation for African buffalo. (PDF 45 kb) [file 12864_2016_3364_MOESM11_ESM.pdf]

Supplementary Table 6: Summary of function annotation for African buffalo.

	Number	Percentage (%)
**Total**	19,296	--
**InterPro**	17,372	90.03
**GO**	13,723	71.12
**KEGG**	14,729	76.33
**Swissprot**	18,477	95.76
**TrEMBL**	18,807	97.47
**Annotated**	18,827	97.57
**Unannotated**	469	2.43
